# Cultural formulation interview: Awareness and attitudes of South African psychiatrists and psychiatry registrars

**DOI:** 10.4102/sajpsychiatry.v26i0.1467

**Published:** 2020-10-23

**Authors:** Dijeng J. Mabaso, Carla Kotze

**Affiliations:** 1 Department of Psychiatry, School of Medicine, Faculty of Health Science, Weskoppies Psychiatric Hospital, University of Pretoria, Pretoria, South Africa

**Keywords:** attitudes, Cultural Formulation Interview, psychiatrists, culture, mental health, formulation

## Abstract

**Background:**

The Cultural Formulation Interview (CFI) includes a set of 16 questions formulated for clinicians to obtain information about cultural influences on an individual’s clinical presentation during a mental health assessment. The CFI is a newly formulated interview that has been evaluated in various localities, but not in South Africa.

**Aim:**

We assessed the awareness and attitudes of South African psychiatrists and psychiatrists in training towards the CFI and its use in their patient care.

**Setting:**

Participants were recruited via the South African Society of Psychiatrists (SASOP) database and were invited to complete an online questionnaire.

**Methods:**

Certain demographic aspects of psychiatrists and psychiatrists in training were compared with regard to their awareness of and attitudes towards the CFI.

**Results:**

Of the 75 participants who completed the questionnaire, only 46.7% (*n* = 35) were aware of the CFI, and of these, only 5.3% (*n* = 4) used the CFI. There was generally a positive attitude towards the CFI with the majority of the participants agreeing that the CFI is easy to understand and relevant in clinical practice; however, none of the results showed statistical significance. Most participants who were aware of the CFI (89%, *n* = 31) were of the opinion that the CFI would prolong their patient assessment time.

**Conclusion:**

Most participants had a positive attitude towards the CFI. The negative response regarding the CFI prolonging patient assessment time could be a potential barrier in its clinical application. This study is an essential first step for further research into the CFI and its use in SA and contributed towards improving awareness of the CFI.

## Introduction

The world has become a melting pot of cultures. Immigration and emigration, by choice or through displacement, for economic and political reasons, destabilise cultural mindsets and require adaptation to new environments, values and ways of life. The African view of mental illness and health currently encompasses a wide spectrum of systems – from ancestors and folk belief in witchcraft to modern medical science. All these systems function simultaneously within the African culture and within some individuals. As a multicultural society with a predominantly African culture, South Africa provides a great opportunity for mental health professionals to develop cultural sensitivity and apply it in practice.^1,2^

Cultural competence refers to the knowledge and skills that are needed to function effectively and appropriately in culturally diverse situations. Cultural competence is a framework of practice for culturally diverse settings that can be applied when working with issues of mental health. Some of the critiques against it are that it focusses on providers and disregard the patients and their communities, and it draws on static notions of cultures. There is increasing acknowledgement of cultural diversity in mental health systems. One such example is the inclusion of the Cultural Formulation Interview (CFI) in the *Diagnostic and statistical manual of mental health disorders*, Fifth Edition (DSM-5).^3,4^

The CFI provides a systematic review of the individual’s cultural background and is a positive step towards exploring the cultural identity, conceptualisation of illness, psychosocial stressors and vulnerabilities of patients. It considers the role of the cultural context in the expression and evaluation of symptoms and dysfunction, and the effect that differences may have on the relationship between the individual and the clinician. Interventions that introduce clinicians to patient cultural views may increase patient participation throughout the interview. They also enhance clinician–patient information exchange, interpersonal rapport and preferences to treatment. Therefore, cultural formulation is an essential component of any comprehensive assessment, because culture can shape every aspect of patient care in psychiatry.^5,6^

Past studies focussed on the Outline for Cultural Formulation (OCF) and the challenges of implementing OCF in clinical practice and in research.^7,8,9,10,11,12^ The OCF was operationalised by the formulation of the CFI with the intention of enhancing the cultural validity of diagnostic assessment, facilitating treatment planning and promoting individual participation and satisfaction.^5,13^

Before publication of the DSM-5, considerable efforts went into identifying possible barriers to implementing the CFI in practice. The CFI was tested for feasibility, acceptability and perceived utility amongst patients and clinicians. Clinicians identified barriers regarding uncertainty about questions on culture, redundancy, patients for whom the CFI may not work well, extra time, length, training and experience needed to deploy the CFI. The length of time to complete the interview was seen as a potential barrier in the feasibility and acceptability of these interviews in widespread clinical practice.^6^ Cultural Formulation Interview field trials and cross-cultural evaluations have been conducted that involved training and use of the CFI. A study that included 318 patients and 75 clinicians from 6 countries, including Canada, India, Kenya, the Netherlands, Peru and USA, supported the feasibility, acceptability and clinical utility of the CFI. Clinician’s attitudes towards the CFI improved significantly after the first interview, and subsequent interviews also required less time. Clinicians with culturally diverse patients rated the CFI more positively.^14,15^

South African mental health practitioners have attempted to describe culture-bound syndromes and their effect on diagnosis. They have also investigated the relationship between cultural beliefs and the content of delusions in Xhosa-speaking patients with schizophrenia and highlighted the value of using culturally sensitive assessment tools, but no studies about the use of the CFI in practice have been performed in South Africa.^16,17^ Despite South Africa’s cultural heritage, there is little evidence that local psychiatrists are aware of the revised CFI, and it is unknown if the CFI will be widely acknowledged and used by various mental healthcare providers in South Africa.

The CFI has been discussed in the context of the South African Society of Psychiatrists (SASOP) guidelines for the integration of spirituality in the approach to psychiatric patients. These guidelines highlight the differences between religion, spirituality and culture, with religion being considered as a component of an integrated cultural whole. The guidelines recommended the integration of spirituality and cultural competencies in psychiatric training and practice.^18^ However, a literature search with Pub Med/MEDLINE, Medscape and Google Scholar could not find any studies that focussed on the use of the CFI in South Africa. To lay the foundation for research into this area, this study aimed to assess and compare the awareness and attitudes of psychiatrists and psychiatry registrars (psychiatrists in training) in South Africa towards the CFI. As lack of awareness or negative attitudes towards the CFI may hinder its effective implementation and use in clinical practice, this study is an essential first step for further research, to improve awareness of the CFI and to encourage its use.

## Methods

An exploratory, quantitative, cross-sectional survey was conducted amongst South African psychiatrists and psychiatry registrars by using Survey Monkey, an online web browser-based survey software application.

Over a 6-month period, from February to August 2016, the survey was sent out per email to all psychiatrists on the SASOP database. South African Society of Psychiatrists is a non-profit company with the aim to promote, maintain and protect the interest of the discipline of psychiatry and its members. The survey was sent out on three occasions, every second month, during the 6-month period to an estimated 500 individual email addresses.

Convenience sampling was used and participation was open to psychiatrists registered with the Health Professions Council of South Africa (HPCSA), practising in either the private or the public sector. Psychiatry registrars in their 4-year training programme and registered with the HPCSA could also participate. Medical officers not recognised by the HPCSA for training in psychiatry were excluded. The participants who did not complete the full questionnaire were also excluded.

The variables measured include: socio-demographic factors (including area of practice and years of experience), awareness and attitudes towards the CFI and if it is applied in clinical practice.

### Questionnaire

The self-administered questionnaire was specifically designed to measure the attitudes of psychiatrists and registrars towards the CFI. Some of the constructs of this scale were derived from themes proposed by Aggarwal et al. and modified specifically for this study.^6^

As part of pilot testing, the questionnaire was presented at the Department of Psychiatry, University of Pretoria, research meetings for input from consultants and registrars and to refine the item formulation. The questionnaire was a nine-item Likert scale survey with four response categories, i.e. strongly disagree, disagree, agree and strongly agree. Components were rephrased in a closed-ended statement format for simplicity and to save time, to be more specific, to allow similar meanings to be communicated and to maintain consistency in responses to allow comparison between respondents.

After enquiring about the awareness and use of the CFI, the statements in the questionnaire were: (1) It is easy to understand; (2) It might prolong my patients assessment; (3) It is relevant in clinical practice; (4) It is of benefit to my patients; (5) I will try to apply it in my practice; (6) It can assist in formulating a diagnosis; (7) It can assist in formulating a treatment plan; (8) It is good enough to assess cultural factors affecting illness presentation; and (9) Training on how to apply it in practice is necessary.

The response category was compared with different demographic factors. The responses to the questions were dependent on how the questions were phrased; thus agreeing or strongly agreeing to the component did not necessarily indicate a positive response to some of the components. To measure whether the items on this questionnaire all reliably measure the same construct, the internal consistency of the questionnaire was evaluated by carrying out a reliability test by using Cronbach’s alpha. The reliability results showed that each item of the attitude questionnaire had scores of α ≥ 0.7 and all of them summed together scored *α*s = 0.78, indicating acceptable internal consistency. However, when one of the components which had higher scale of *α*s = 0.80 (CFI might prolong assessment time) was removed, this increased the summed *α*s of the other eight components to 0.80. This indicated a good internal consistency for the questionnaire according to Cronbach’s alpha.

Each participant received an email invitation, with a link to the demographic data sheet, study questionnaire and the supplementary CFI questionnaire. A reference webpage, www.psychiatry.org/dsm5 was provided for participants who were interested in understanding the depth of the CFI questionnaire. This gave participants an opportunity to familiarise themselves with the CFI before answering the questionnaire. The CFI recommends the consideration of various cultural aspects when performing psychiatric assessments and formulations and comprises a set of 16 questions. The four domains of assessment are: cultural definition of the problem, cultural perceptions of cause, context and support, cultural factors affecting self-coping and past help-seeking and cultural factors affecting current help-seeking.^13^

### Statistical analysis

For categorical data including age, years of experience, registration category and area of practice, descriptive statistics were used. Data were summarised in cross tables reporting frequencies and percentages. Fisher’s exact test was used to measure associations between attitude and awareness components and socio-demographic factors. Testing was performed at the 0.05 level of significance. The internal consistencies of the attitude items included in the questionnaire were assessed by using Cronbach’s alpha.

### Ethical consideration

Ethical approval was obtained from the Research Ethics Committee of the Faculty of Health Sciences, University of Pretoria (reference number: 190/2015). The study has been structured in accordance with the Declaration of Helsinki (last update: October 2013). An information leaflet was included with information about the study, and this was followed by a statement indicating that completion and submission of the questionnaire affirmed informed consent by the participant. Once the questionnaire had been submitted, the participants could not withdraw from participating. Data were automatically stored electronically within the link and only accessible to the researcher.

## Results

### Respondent characteristics

There was a response rate of approximately 19% with 93 responses received, but only 80.6% (*n* = 75) completed the full questionnaire, of whom 66.7% (*n* = 50) were psychiatrists and 33.3% (*n* = 25) were psychiatrists in training. In terms of gender, 61% (*n* = 46) of participants were female and 39% (*n* = 29) of participants were male.

Of all the respondents, 52% (*n* = 39) practised in both inpatient and outpatient settings, 25.3% (*n* = 19) exclusively in inpatient settings and 22.7% (*n* = 17) exclusively in outpatient settings. For the ages of respondents, years of experience in mental health and area of practice, refer to Table 1.

**TABLE 1:** Age, years of experience and area of practice of respondents.

Characteristics	Number	%
**Age of respondents**		
25–35 years	26	34.7
36–46 years	37	49.3
56–65 years	12	16
**Years of experience**		
< 5 years	18	24
6–20 years	35	46.7
> 20 years	22	29.3
**Area of practice**		
Academic	47	62.7
Private	26	34.7
District	2	2.7

### Awareness and usage of the Cultural Formulation Interview

Of the 75 participants who completed the questionnaire, only 46.7% (*n* = 35) were aware of the CFI; of these only 5.3% (*n* = 4) used the CFI and all 4 were practising in academic settings. Of the 35 participants who were aware of the CFI, 62.9% (*n* = 22) were psychiatrists, 74.3% (*n* = 26) were practising in the public/academic sector, 25.7% (*n* = 9) had < 5 years of experience, 42.8% (*n* = 15) had 6–20 years of experience and 18 (51%) of those spent most of their time in both inpatient and outpatients settings.

### Attitude components

**FIGURE 1:**
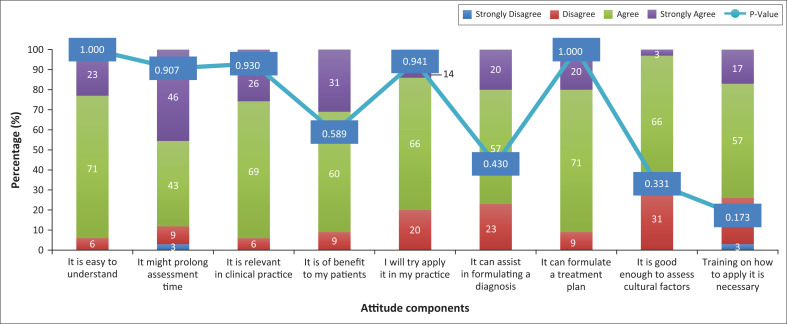
Associations between awareness and attitude components.

Participants who were aware of the CFI (*n* = 35) were mostly in agreement with the attitude questionnaire components. Almost half of the participants (46%, *n* = 16) strongly agreed and 43% (*n* = 15) agreed that the CFI might prolong patient assessment time (Figure 1). However, none of the results showed statistical significance.

### Attitude responses according to the area of practice

**FIGURE 2:**
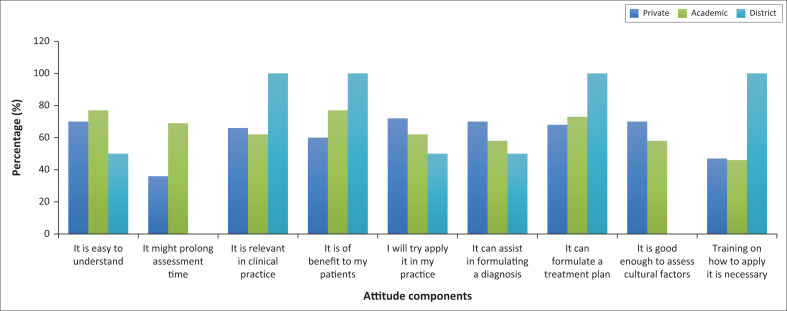
Attitude responses according to area of practice.

Most of the participants in all three areas of practice agreed with most of the attitude components and the results are shown in Figure 2.

Significantly, participants in private (53%, *n* = 25) and district settings (100%, *n* = 2) strongly agreed that the CFI may prolong their patient assessment time compared with participants in academic settings (69%, *n* = 18), who just agreed (*p* = 0.035). Furthermore, participants in private (47%, *n* = 22), in academic (46%, *n* = 12) and in district settings (100%, *n* = 2) agreed that training is necessary (*p* = 0.039).

### Attitude responses according to years of experience

**FIGURE 3:**
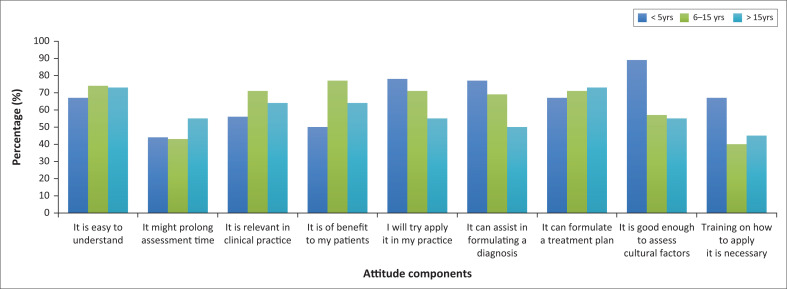
Attitude responses according to years of experience.

Most participants with all three levels of experience agreed with most of the attitude components. The findings did not differ much according to the different years of experience (Figure 3).

Participants with < 5-years of experience (50%, *n* = 9) and those with 6–15 years of experience (49%, *n* = 17) strongly agreed that the CFI would prolong patient assessment time, compared with participants with > 15 years of experience (55%, *n* = 12) who just agreed.

### Attitude responses of psychiatrists versus psychiatrists in training

**FIGURE 4:**
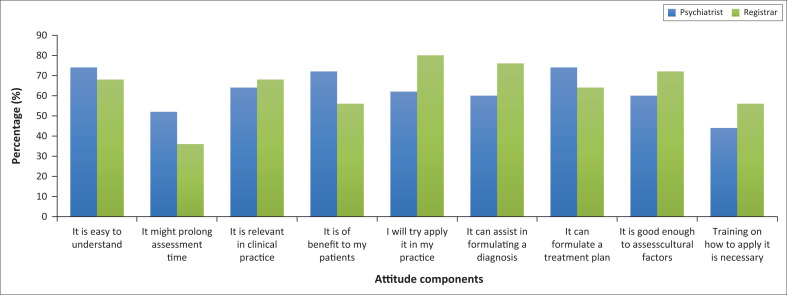
Attitude responses of psychiatrists versus registrars (psychiatrists in training).

Most of the participants in the two categories of speciality agreed with most of the attitude components. There were minor differences between the two categories with the psychiatrists in training agreeing more to apply it in practice compared with the psychiatrists and these findings (Figure 4).

Regarding the CFI prolonging assessment time, 56% (*n* = 14) of psychiatrists in training strongly agreed and 52% (*n* = 26) of psychiatrists just agreed. However, none of the results showed statistical significance.

### Attitude responses according to predominant practice setting

We asked participants to indicate whether they spend most of their time in outpatient or inpatient settings or in both, and also compared these responses. Most participants in all three categories agreed with most of the attitude components. However, 53% (*n* = 9) of participants in inpatient settings and 49 % (*n* = 19) in both settings strongly agreed that the CFI might prolong assessment time compared with 68% (*n* = 23) of participants in outpatient settings. Significantly, 53% (*n* = 9) of participants in inpatient settings strongly agreed that the CFI is relevant in clinical practice compared with 58% (*n* = 11) in outpatient settings and 77% (*n* = 30) in both settings (*p* = 0.041). Forty-seven per cent (*n* = 8) of respondents in inpatient settings agreed, and another 47% (*n* = 8) in outpatients settings strongly agreed that the CFI would benefit their patients.

### Attitude responses according to gender

Regarding the association of the attitude components with gender, most female participants (78%, *n* = 36) agreed that the CFI was easy to understand and 48% (*n* = 14) of male participants agreed that the CFI may prolong assessment time. Of the female participants, 70% (*n* = 32) agreed that the CFI was relevant to clinical practice, 74% (*n* = 34) agreed that their patients would benefit, and significantly 80% (*n* = 37) agreed that they would try to apply the CFI to their practice (*p* = 0.015). Most of the female participants agreed that the CFI would assist in formulating a diagnosis (72%, *n* = 33) and treatment plan (76%, *n* = 35). Male participants agreed that the CFI was good enough to assess cultural factors affecting illness presentation (72%, *n* = 21) and that training on the application of the CFI in practice was necessary (55%, *n* = 16).

## Discussion

According to our knowledge, this is the first study to report on the awareness, use and attitudes of psychiatrists and psychiatrists in training towards the CFI in South Africa. An unexpected finding of this study was that fewer than half (47%) of the respondents were aware of the CFI. Taking into consideration that SASOP released a position statement that culture, religion and spirituality should be considered in the current approach to the local practice and training of specialist psychiatrists, the expectation was that more participants will be aware of this important instrument.^18^

It has been reported that awareness of the CFI amongst healthcare providers was low before the CFI was incorporated into the DSM-5 manual. Studies assessing the feasibility and use of the CFI may serve a dual purpose of creating awareness of the CFI amongst participants and facilitate future use of the CFI in clinical practice.^6,14^ In this way, the current study created an awareness of the CFI amongst respondents who were not aware of the instrument. A recent update about the CFI indicated that the CFI has been evaluated in many countries, but not in South Africa. In the USA, Canada, Kenya, Peru, the Netherlands, India and Mexico, it was generally found to be clinically acceptable and useful in varied settings.^19^

In our study, it was found that despite knowing about the CFI, very few participants used the CFI for clinical assessments. The various reasons that may exist for such a behaviour were not investigated in our study. Previously, it was reported that clinicians might not be motivated or buy into the use of the CFI or may transition into regular clinical assessment after conducting the CFI, defeating the purpose of the CFI.^6^ The CFI serves to identify unique cultural aspects that may influence diagnosis and treatment. It is possible that some clinicians still do not acknowledge the role of cultural context on diagnosis and treatment of various mental health-related conditions. This can hinder the use of the CFI in clinical settings, but research to confirm this, specifically in South Africa, is not available.

In our study, psychiatrists in academic settings had the greatest awareness of the CFI and formed the majority of respondents. These psychiatrists possibly had better access to information in academic hospitals as the DSM-5 and the CFI were frequently used in academic teaching and training in South Africa, but it might also be biased because of the higher number of respondents from the academic setting.

In the DSM-5 field trial, participants found the CFI useful with respect to diagnosis, treatment planning and understanding the patient’s situation, including the role of culture in mental illness.^14^ This was found to be similar in our study where participants who were aware of the CFI showed positive responses towards eight of the nine attitude questionnaire components, including those that asked whether they found the CFI easy to understand and relevant in clinical practice.

Before the CFI was incorporated into the DSM-5, time needed to use the CFI in practice was seen as an implementation barrier, and lack of time has previously been described as a barrier to adherence to guidelines.^6,20,21^ In our study, there was a similar finding that despite the positive responses to most of the attitude questions, most participants strongly agreed that the CFI would prolong their patient assessment time. However, in a recently published mixed-method field trial, the administration of the CFI resulted in shorter follow-up interviews. The long-term benefits of using the CFI improved clinicians’ feasibility ratings, and clinicians should be made aware of this possible benefit to improve implementation of CFI use in clinical practice.^14^

Most of the participants, irrespective of whether they practise in academic, private or district settings, agreed that physicians needed training to learn how to apply the CFI in practice. From the DSM-5 CFI field trials, it was found that clinicians across countries preferred case-based behavioural simulations in cultural competence training and this will need further investigation in implementation in the South African setting.^22^ It has been shown that as little as 1 h of training on the CFI can improve clinicians’ ability to work with culturally diverse patients.^19^

South African psychiatrists in inpatient settings strongly agreed that the CFI is relevant in clinical practice compared with psychiatrists spending more time in outpatient settings, who simply agreed that the CFI was relevant. The reason for this difference was not investigated as part of this study, but the American Psychiatric Association has stated that the revised CFI is applicable and useful in all clinical settings.^13^ In this study, it was also found that female psychiatrists were more willing to apply the CFI in their clinical practice than males. Although the reason for this finding was not investigated, it is likely influenced by the sample that consisted predominantly of females, similar to previous studies.^6,21,22,23^

## Limitations

There was a poor response rate to the online survey, with fewer responses than initially anticipated. The smaller sample sizes might have affected the statistical significance of some of the results. In a study exploring specialists’ response rates to web-based surveys, 16.9% of psychiatrists were found to be non-responders in the follow-up survey. The contributing factors identified were survey burden, too many survey requests and lack of time to complete them and no interest or seeing no benefit in completing the survey.

The same reasons could have contributed to the poor response rates seen in our study.^24^

Previous studies have shown that the attitudes of clinicians can be influenced by training, and the participants in our study were not trained to apply the CFI and most of them have not applied the CFI in clinical practice. However, the respondents had the opportunity to look at the CFI through the reference website that was provided to gain a better understanding of the questionnaire. Despite the limitations identified, our work improved the awareness of the CFI in South Africa and contributes to research about the CFI and potential barriers to its implementation.

## Recommendations for future research

In our study, we did not give respondents an opportunity to use the CFI before giving a response. Future research could give an indication of whether the participants still strongly agree that the CFI prolongs assessment time after they have an opportunity to use it. A qualitative approach is recommended to try and establish reasons for the differences seen amongst the various groups in our study and to get more detailed information about attitudes towards cultural influences in psychiatric assessments and use of tools such as the CFI. The focus of future research could be to establish the preferred training methods for our study population in the use of this type of instrument.

## Conclusion

Lack of awareness and a negative attitude towards interventions can hinder the effective implementation of the CFI in clinical practice. The CFI is designed to be used by clinicians in any setting to assist with the gathering of essential data to produce a cultural formulation. Overall, the participants in our study had a positive attitude towards the CFI. The attitude findings did not differ much when compared according to demographics. However, the overall negative response regarding the CFI prolonging patient assessment time could still be a potential barrier in its clinical application. It will be important for future advocacy or research efforts to make clinicians aware of the potential benefits of incorporating the CFI into their assessments, whilst also informing them that it has been found that with practice, the CFI takes only approximately 20 min to complete. The CFI can be an effective way to initiate cultural assessments, and preliminary evidence indicates that the CFI can improve clinical communication and enhance clinician–patient rapport.^19^ This study is an essential first step for further research into the CFI and its use in SA and contributes towards improving awareness of the CFI amongst South African psychiatrists.
